# Characterization and Genetic Mapping of Black Root Rot Resistance in *Gossypium arboreum* L.

**DOI:** 10.3390/ijms22052642

**Published:** 2021-03-05

**Authors:** Iain W. Wilson, Philippe Moncuquet, Marc Ellis, Rosemary G. White, Qian-Hao Zhu, Warwick Stiller, Danny Llewellyn

**Affiliations:** 1 CSIRO Agriculture and Food, GPO Box 1700, Canberra, ACT 2061, Australia; Philippe.moncuquet@csiro.au (P.M.); Rosemary.White@csiro.au (R.G.W.); Qianhao.Zhu@csiro.au (Q.-H.Z.); Danny.Llewellyn@csiro.au (D.L.); 2 133 Route de Beauregard, 74540 Gruffy, France; mhe.mex@gmail.com; 3 CSIRO Agriculture and Food, Locked Bag 59, Narrabri, NSW 2390, Australia; Warwick.Stiller@csiro.au

**Keywords:** *Thielaviopsis basicola*, *Berkleyomyces rouxiae*, black root rot, disease resistance, *Gossypium arboreum*, transcriptome analysis, asiatic cotton

## Abstract

Black root rot (BRR) is an economically important disease of cotton and other crops, especially in cooler regions with short growing seasons. Symptoms include black discoloration of the roots, reduced number of lateral roots and stunted or slow plant growth. The cultivated tetraploid Gossypium species are susceptible to BRR. Resistance to BRR was identified in *G. arboreum* accession BM13H and is associated with reduced and restricted hyphal growth and less sporulation. Transcriptome analysis indicates that BM13H responds to infection at early time points 2- and 3-days post-inoculation, but by day 5, few differentially expressed genes are observed between infected and uninfected roots. Inheritance of BM13H resistance to BRR was evaluated in an F_6_ recombinant inbred population and shows a single semi-dominant locus conferring resistance that was fine mapped to a region on chromosome 1, containing ten genes including five putative resistance-like genes.

## 1. Introduction

Black root rot (BRR) is a seedling disease of cotton caused by a soil-borne filamentous, hemibiotrophic fungus previously known as *Thielaviopsis basicola,* but recently reclassified into two species, *Berkleyomyces basicola* and *B. rouxiae* [1]. BRR is considered a significant threat to cotton and other crops in Australia, especially in the southern regions with shorter production seasons. The fungus survives in the soil primarily as chlamydospores [2], which upon germination invade the roots either directly or through wounds, root hairs or epidermal cells of young seedlings, and then spreads throughout the cortex. Invasion of the root is associated with cooler soil temperatures and occurs during the first 2–8 weeks of cotton growth, whereas older roots appear to be more resistant to infection [3]. The seedling disease characterized by dark brown or black discoloration of the roots as a result of the rotting of the cortex [4]. Diseased cotton plants show stunted or slow growth early in the season compared to uninfected plants, causing delayed flowering or maturity that can result in up to a 46% decrease in seed cotton yield [5]. In addition to the direct effect of BRR infection, lesions caused by the fungus may facilitate infection by other cotton seedling pathogens such as *Rhizoctonia*, *Pythium,* and *Fusarium* [6] and in North America significant interactions have been observed between *T. basicola* and the root-knot nematode *Meloidogyne incognita* [7].

BRR is widespread throughout Australian cotton-growing areas and through the production of chlamydospores BRR can survive in the soil for many years once established [2]. Management of this disease is currently accomplished by a range of measures that are designed to limit fungal inoculum levels from building up in the soil, and include crop rotation, flooding and fungicide seed treatments [6]. Improved host plant resistance to BRR is required for the long-term control of this disease in affected fields. However, large-scale screening of tetraploid cotton has not identified significant sources of resistance to BRR [8,9,10] but two diploid cotton accessions have been found with differing degrees of BRR resistance [11]. *Gossypium arboreum* accession PI 1415 had reduced root necrosis compared to susceptible checks at moderate levels of infection, but resistance was overcome at high inoculations levels. Whereas *G. herbaceum* accession ‘A20’ was found to possess high levels of BRR resistance even at high inoculation levels [11]. A quantitative trait loci (QTL) analysis of an interspecific F_2-3_ population derived from PI 1415 and A20 identified the presence of at least three loci that conferred increased resistance to BRR [12].

In the present study, BRR was shown to be caused by *B. rouxiae* and BRR resistance identified for *G. arboreum* accession BM13H [13] was found to be associated with reduced and restricted hyphal growth in root tissue and limited sporulation as compared to the susceptible *G. arboreum* accession Yuenanzhongmian (YZ). Transcriptome analysis of BM13H and YZ roots infected with *B. rouxiae* revealed very contrasting responses with BM13H responding early to infection but the number of transcriptional changes reduced over time, whereas in YZ, large changes were observed starting at day 3 and then increasing dramatically with the duration of infection. Inheritance of BM13H resistance to BRR was analyzed in an F_6_ BM13H x YZ recombinant inbred (RIL) population and was found to be conferred by a single semi-dominant locus that was fine mapped to a ~128.5 kbp region on chromosome 1, a region containing ten annotated genes including five putative resistance-like genes, including two; *Ga01G2671* and *Ga01G2672* that are differentially expressed between uninfected BM13H and YZ roots.

## 2. Results

### 2.1. Classification of BRR Fungal Species Infecting Australian Cotton

A molecular phylogenetic analysis by Nel et al. [1] of geographically isolated fungal isolates that cause BRR disease in a number of countries, showed that what was previously classified as *T. basicola* is in fact two species that were renamed; *B. basicola* and *B. rouxiae*. To determine which species is associated with BRR of cotton in Australia, DNA was isolated from a single spore culture of the fungal isolate WTB-001 used throughout this study. The isolate was recovered from a field-grown cotton plant displaying severe BRR symptoms. Amplification and sequencing of the internal transcribed spacer (ITS) region located between the 18S and 28S rRNA genes using the primers ITS1 and ITS4 [14] (Table S1) revealed that this isolate perfectly matched *B. rouxiae* (Figure S1). Analysis of previously deposited ITS sequences in GenBank from Australian BRR-infected cotton samples such as HM031125 [15] and an extensive collection of cotton BRR isolates from different cotton-growing regions in Australia carried out by Dr D. Le (NSW DPI, personal communication) also showed that all BRR-causing isolates from cotton were *B. rouxiae*, and not *B. basicola*, indicating that this species is most likely the only cause of BRR of cotton in Australia.

### 2.2. Microscopic Characterization of BRR Infection in BM13H and YZ

**Figure 1:**
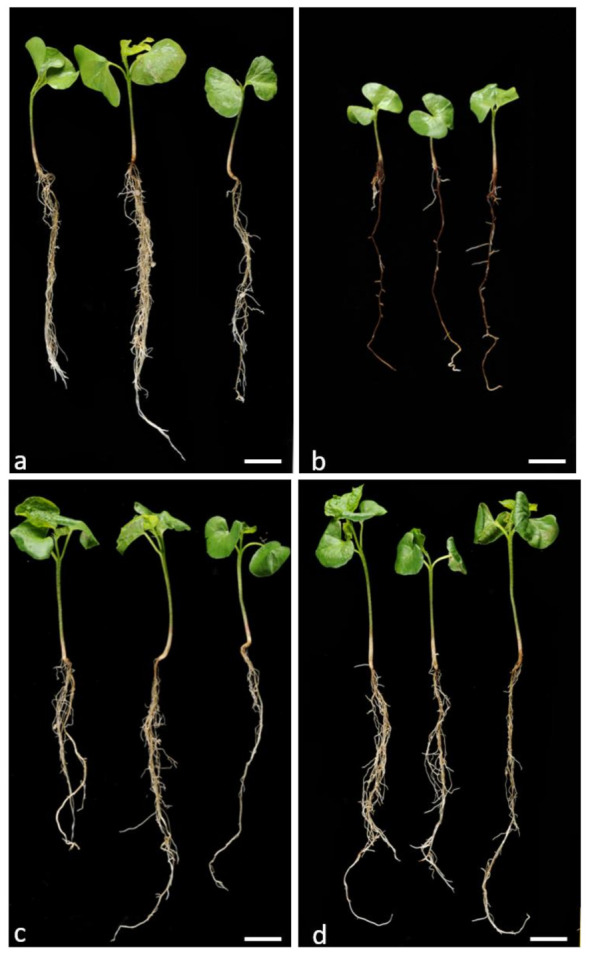
Parental phenotypes of the black root rot (BRR)-susceptible YZ uninfected (**a**) and infected (**b**) and resistant BM13H uninfected (**c**) and infected (**d**) seedlings 25 days after germination in soil without (**a**,**c**) and with (**b**,**d**) BRR spores. Bars = 2 cm.

**Figure 2:**
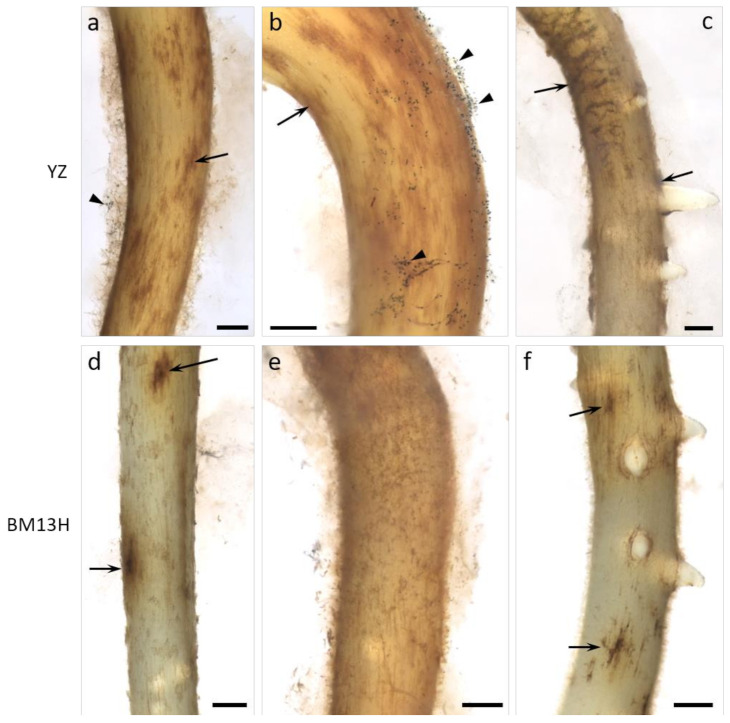
Surface views of roots from susceptible (YZ—**a**–**c**) and resistant (BM13H—**d**–**f**) *G. arboreum* seedlings 5 days after inoculation with BRR spores in sterile culture. The roots became colonized in both accessions (black and brown patches, small arrows), but fungal growth is much more advanced on YZ roots, with abundant spores (arrowheads) already formed and more extended root blackening evident. Some BM13H roots support considerable fungal growth (**e**), but little sporulation. Bars = 0.5 mm.

**Figure 3:**
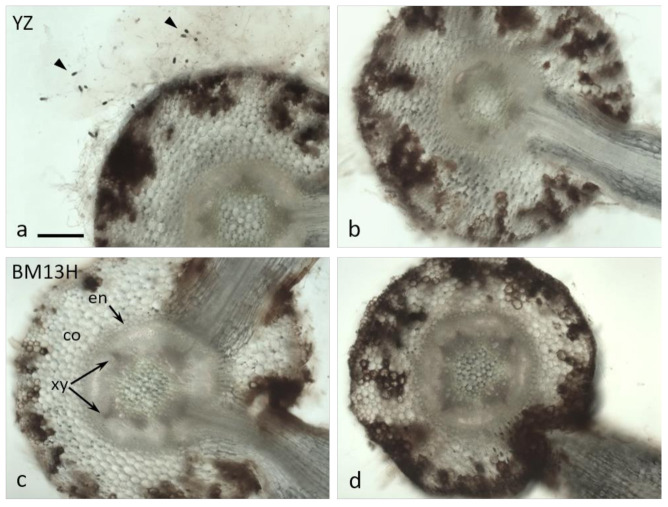
Cross-sections of roots from susceptible (YZ—**a**,**b**) and resistant (BM13H—**c**,**d**) *G. arboreum* seedlings 7 days after inoculation with BRR spores in sterile culture. The epidermis and cortex became colonized to varying degrees in both accessions, with dark fungal hyphae and cortical blackening generally more extensive in YZ roots. Abundant spores (arrowheads in **a**) are evident emerging from fungal mycelium on YZ roots. co = cortex, en = endodermis, xy = xylem poles (2 of 5 indicated in **c**) Bar in a for **a**–**d** = 0.2 mm.

A systematic survey for BRR-resistant germplasm using a glasshouse bioassay with soil taken from fields containing the disease, revealed no resistance in a wide range of the tetraploid *G. hirsutum* and *G. barbadense* accessions (Dr W. Stiller personal communication), similar to that reported by Wheeler et al. [10] in the U.S.A. However, two *G. arboreum* accessions with contrasting levels of BRR resistance were identified in this screen; BM13H which displayed few obvious symptoms, and YZ which displayed symptoms similar to that observed in all *G. hirsutum* cultivars. Under controlled infection conditions in a glasshouse bioassay with the *B. rouxiae* isolate WTB-001 (250 colony forming units (CFU)/gram), BM13H plants were largely unaffected. Whereas YZ plants were stunted, had few lateral roots and almost complete blackening of the roots (Figure 1). Under sterile culture conditions, microscopic examination of the infection process in BM13H and YZ plants showed that fungal hyphae could be detected and associated with the taproots by 3 days post inoculation (dpi) and produced spores by 5 dpi on the susceptible YZ (Figure 2a–c). Hyphae were much less abundant on the surface of BM13H roots, infection sites were smaller, and spores were rarely seen until 7–10 dpi (Figure 2d–f and Figure 3c,d).

**Figure 4:**
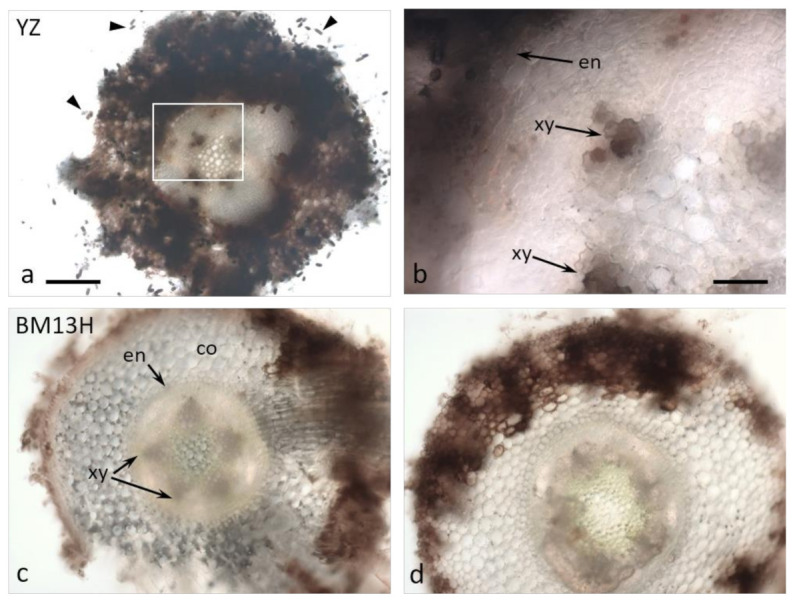
Cross-sections of roots from susceptible (YZ—**a**,**b**) and resistant (BM13H—**c**,**d**) *G. arboreum* seedlings 10 days after inoculation with BRR spores in sterile culture. (**a**,**b**) Abundant spores emerge from the surface of YZ roots (arrowheads in **a**), the cortex (co) is very heavily colonized, and black fungal hyphae commonly penetrate through the endodermis (en) and colonize the xylem (xy). **b** = enlarged area outlined in **a**. (**c**,**d**) There is less fungal colonization of BM13H roots and penetration through the endodermis is rare. co = cortex, en = endodermis, xy = xylem poles (2 of 5 indicated) Bar in a for **a**, **c**, **d** = 0.2 mm, bar in **c** = 50 mm.

Cross-sections taken through root segments showing dark surface discoloration at 7 dpi revealed considerable invasion into the root cortex by *B. rouxiae* hyphae (Figure 3). In sterile culture, more abundant hyphae were evident on YZ roots (Figure 4a) with abundant spores (arrowheads in Figure 4a). Colonization of the cortex was variable in both cultivars but was generally shallower in BM13H roots (e.g., Figure 3c) than in YZ roots (Figure 3b), although some sections of BM13H roots showed deep penetration of the black fungal hyphae (Figure 3d).

By 10 dpi, the hyphae had penetrated the endodermis in some replicates of the susceptible YZ cultivar (Figure 4a,b), and either the hyphae themselves or the root response appeared to block the xylem (Figure 4b, arrows). None of the resistant BM13H roots appeared to contain fungal hyphae within the stele, although the outer cell layers of epidermis and cortex were often strongly colonized to the endodermis (Figure 3d and Figure 4c,d).

**Figure 5:**
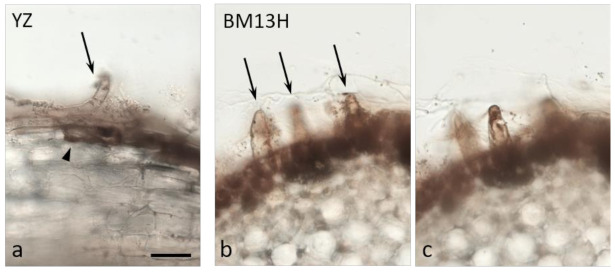
Cross-sections of roots from susceptible (YZ—**a**) and resistant (BM13H—**b**,**c**) *G. arboreum* seedlings 10 days after inoculation with BRR spores in sterile culture. Epidermal cells (arrowhead in a) and root hairs (arrows) are heavily colonized with dark fungal hyphae. (**b**,**c**) are different optical sections of the same root cross-section. Bar in a for **a**–**c** = 50 mm.

Both root hairs and epidermal cells were penetrated by hyphae in both cultivars (Figure 5), and infection generally occurred earliest in the zone of young, fully elongated root hairs.

BM13H is therefore not immune to *B. rouxiae* but seems to reduce and restrict fungal growth such that by eye, infection is not visible on the roots, and growth is not significantly affected (Figure 1c,d).

### 2.3. Comparative Transcriptome Analysis in BM13H and YZ Accessions

To explore the gene expression associated with response to BRR infection in resistant BM13H and susceptible YZ, RNA-seq analysis was performed on root tissue taken from uninfected and infected plants at 2, 3, 5, 7, and 10 dpi. On average, each of the 60 samples sequenced had over 24 million clean paired-end reads, and the average Q30 percentage (sequences with sequencing error rates lower than 0.03%) was 93.3%, indicating the high quality of the RNA-seq data. Between 71% and 82% of clean reads from these samples could be aligned to the *G. arboreum* reference genome [16] (Table S2). When comparing infected to uninfected root tissue in each accession there was a difference in the number and type of differentially expressed genes (DEG) observed (Table 1, Tables S3 and S4). BM13H had 53 DEGs at 2 dpi (43 upregulated, 10 downregulated) that increased to 133 (59 upregulated, 74 downregulated) by 3 dpi, but this then decreased to 8 DEGs (5 upregulated, 3 downregulated) at 5 dpi and only two DEGs at 7 and 10 dpi (Table 1, Table S3). Meanwhile, YZ had 8 DEGs at 2 dpi (3 upregulated, 5 downregulated), but this number increased from 72 (57 upregulated, 15 downregulated) at 3 dpi to 1969 (1606 upregulated, 363 downregulated) at 5 dpi, 2321 (1660 upregulated, 661 downregulated) at 7 dpi and 6332 (3740 upregulated, 2592 downregulated) at 10 dpi (Table 1, Table S4). To look at accession-based (not infection dependent) gene expression differences, all uninfected samples were compared between BM13H and YZ, and 3197 DEGs were observed (Table S5). To identify biological processes and functions associated with the different transcriptional responses of BM13H and YZ, all BM13H infected and uninfected samples were compared directly to YZ infected and uninfected samples, respectively, and Gene Ontology (GO) enrichment analysis was performed on genes specific to infection and uninfected expression differences. Infection-specific DEGs associated with BM13H (Table S6) were enriched in 16 biological processes (FDR < 0.01), including carbohydrate metabolic processes, starch biosynthesis and plant cell wall organization (Table 2, Figure S2). The DEGs associated with YZ infection (Table S7) were enriched for 102 biological processes with major enriched terms for regulation of signaling and the immune system, defense and stress responses, and responses to the hormone salicyclic acid (Table 3, Figure S3). There were no enriched GO terms found for the DEGs between uninfected BM and YZ root samples.

### 2.4. Inheritance of BBR Resistance

To understand the genetics of the resistance observed in BM13H, crosses were made between BM13H and YZ. In a glasshouse based BRR infection assay of 51 F_1_ seedlings in soil containing 250 CFUs of BRR spores per gram of soil, the average BRR disease rating (0–100%) based on the percentage of necrotic root tissue [12] of the roots was 39% for the F_1_ plants compared to 0% for BM13H (17 plants) and 90% for YZ (18 plants) indicating an intermediate level of resistance. An F_1_ plant was self-pollinated and 263 F_2_ progeny generated and infected with WTB-001. The infection results at 25 dpi indicate a bimodal infection level with approximately 25% of plants with a root infection percentage of less than 30% (Figure S4). To aid the genetic characterization of the resistance an F_6_ recombinant inbred line (RIL) population from a BM13H x YZ cross was developed by single seed decent from the first 158 individuals from the F_2_ population. Each F_6_ line was subjected to at least two independent assays (two pots per assay, ~eight plants per pot) and the final disease score for each line was the average of the disease rating from ~32 plants. Each assay included four pots of parental plants (~eight plants per pot) and over the course of the assays the disease scores were 1% for BM13H and 88% for YZ lines. The distribution of infection percentages in the RIL population had a clearer bimodal infection distribution than in the F_2_ population, with most lines falling either into the ≤30% or ≥80% necrotic root ranges (Figure S5).

### 2.5. Genetic Mapping of Black Root Rot Resistance

A total of 178 genetic markers (Tables S8–S10) comprising simple sequence repeat (SSR), cleaved amplified polymorphic sequences (CAPS), and single nucleotide polymorphism (SNP)-based markers (Kompetitive Allele Specific PCR (KASP) or Fluidgm) were analyzed on the 158 RIL F_6_ lines. After linkage analysis, the markers were assembled into 17 linkage groups with a total length of 1294 cM, and linkage groups were assigned to chromosomes using the DNA marker sequences matches to the genome assembly of the *G. arboreum* cultivar Shixiya1 (SXY1) [16] (Figure S6).

**Figure 6:**
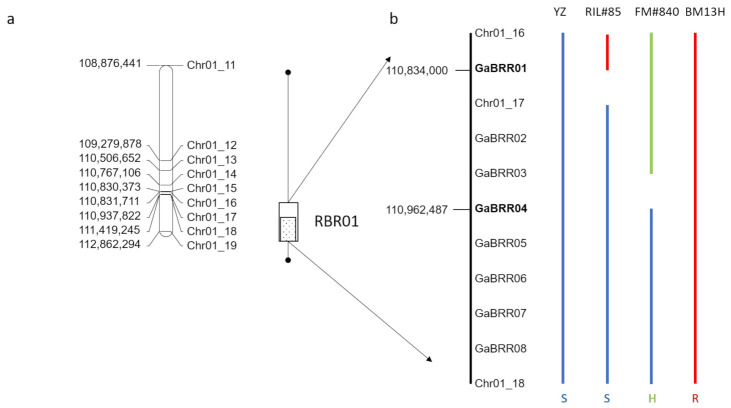
Mapping of the BRR resistance locus RBR01. (**a**) Single marker analysis associated resistance with markers Chr01_11 to Chr01_19 (line with closed circles at end), and a single QTL was detected between markers Chr01_17 to Chr01_18 (dotted box). Scoring the phenotype as a simple trait indicated the interval was between Chr01_16 and Chr01_18 (open box). (**b**) Fine mapping of the region with key recombinants reduced the interval to between GaBRR01 and GaBRR04 markers (bold), S = susceptible (Blue), H = Heterozygous (green) and R- resistant (red).

Single marker analysis, using a nonparametric mapping test equivalent to a one-way analysis of variance [17], found nine markers on chromosome 1 (Chr01_11 to Chr01_19) associated with BRR resistance (*p* ≤ 0.001) (Figure 6A). A single QTL (BRR01) above the permutation threshold, was detected for BRR resistance by multiple QTL model analysis [17] between markers Chr01_17 and Chr01_18 which explained 98% of the phenotypic variation (Figure 6a). The QTL analysis, therefore, indicated that resistance was conferred by a single locus that we call ‘Resistance to *Berkleyomyces rouxiae* 1′ (RBR01). The RIL disease data were then re-analyzed such that plants with roots with disease ratings of ≤30% were designated as resistant, roots having a disease rating of ≥70% were designated as susceptible, and families that possessed plants with infection scores that included at least one resistant and one susceptible plant were designated as heterozygous. Using this simplified scoring system, RBR01 was mapped between the markers Chr01_16 and Chr01_18 (Figure 6a), which is 0.9 cM in genetic distance, and physically covers 587,500 bp (110,831,711-111,419,245) based on the SXY1 genome [16].

### 2.6. Fine Mapping of BRR01

**Figure 7:**
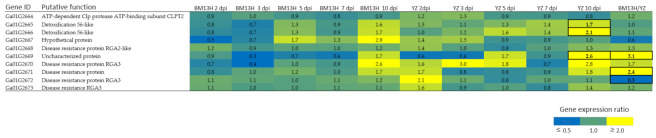
Gene expression ratios of 10 annotated genes in the BRR01 mapping interval, gene expression values are fold-change ratio values (infected/uninfected), statistically significant DEG values are outlined.

To reduce the size of the genetically defined interval, two new populations were developed from RILF6#59 and RILF6#141 families that were found to be segregating for BRR resistance and were scored as heterozygous for the Chr01_16, Chr01_17 and Chr01_18 markers (Table S11). Eight hundred RILF_6_-_7_ plants from each family were planted and genotyped for the Chr01_16 and Chr01_18 markers, and eleven plants with recombination between the two markers were identified (Table S11). These plants were self-pollinated and their progeny tested for BRR resistance. A further eight KASP markers (designated GaBRR01 to 08) were developed in the interval between the Chr01_16 and Chr01_18 markers (Table S8) and used to genotype the original F_6_ recombinants and the eleven fine mapping recombinants (Table S11). The results indicate that the BRR01 locus was located between GaBRR01 and GaBRR04, a distance of 128,488 bp (Figure 6b). This interval has 10 annotated genes *Ga01g2664* to *Ga01g2673* that includes five putative disease resistance like genes (*Ga01G2668*, *Ga01G2670*, *Ga01G2671*, *Ga01G2672*, *Ga01G2673*) (Figure 7).

Analysis of the expression profiles of the ten genes in the mapping interval based on the RNA-seq dataset in Section 2.3 ‘Comparative Transcriptome Analysis in BM13H and YZ accessions’, revealed that only three of those genes were differentially expressed between infected and uninfected roots, during the period of infection analyzed. *Ga01G2665*, *Ga01G2666* and *Ga01G2671* were differentially expressed in YZ at 10 dpi (Figure 7). However, three genes (*Ga01G2669*, *Ga01G2671*, *Ga01G2672*) were differentially expressed between BM13H and YZ when only uninfected roots samples were compared (Figure 7, Table S5). *Ga01G2669* was expressed 3.4-fold higher in BM13H, *Ga01G2671* was expressed 2.3-fold higher in BM13H, and *Ga01G2672* was expressed 3-fold higher in YZ.

## 3. Discussion

BRR has been found in a wide range of economically important plant species, both indigenous and exotic in Australia [8], but the BRR pathogen is thought to be introduced as it has not been identified from undisturbed Australian soils [6]. Comparisons of the ITS sequence from the fungal isolate WTB-001 and other Australian cotton samples infected with BRR [15] indicate that the newly recognized species *B. rouxiae* is the causative agent of BRR in Australian cotton. In addition, *B. rouxiae* has been identified as the cause of BRR on lettuce in Japan [18], implying that the species has a wide host range, although it may show some degree of host specificity. Nevertheless, this wide host range could be useful for the evaluation of diverse mechanisms of resistance that could benefit cotton improvement.

In the absence of any known resistant germplasm in cultivated *G. hirsutum* and *G. barbadense* [12], the impact of the disease on the Australian cotton industry is currently limited solely by a range of cultural practices such as rotation of non-host crops and fungicide application that attempt to limit the build-up of inoculum levels in the soil [6]. Therefore, improved host plant resistance to BRR is needed for the long-term control. Niu et al. [12] identified strong resistance to BRR in the *G. herbaceum* accession ‘A20’ to an T. *basicola* isolate, and QTL analysis revealed that resistance was genetically complex involving at least three loci (BRR5.1, BRR9.1 and BRR13.1) that together explained approximately 40% of the total resistance observed. We identified strong resistance in the *G. arboreum* accession BM13H to a *B. rouxiae* isolate. BM13H is also a diploid cotton with an A-genome, but in this accession, resistance was due to a single locus called RBR01 on Chromosome 1. As none of the QTL identified in *G. herbaceum* [12] are located on Chromosome 1, RBR01 represents a new BRR resistance locus in cotton. 

The surface colonization of cotton roots by BRR [19,20] and the physiological consequences associated with seed cotton yield reductions from heavy infestations [5] have been described several times. Our analysis shows that the progress of hyphal invasion into susceptible roots of *G. arboreum* is very similar to that in tobacco, outlined in several earlier reports [21,22,23]. Indeed, the invasion process and sequence of disease progression appear similar in several other species including bean, pansy and lupin [24,25,26]. When comparing the infection process between YZ and BM13H, the initial stages of invasion into the root epidermis and root hair cells appeared similar (Figure 5), with both cell types harboring abundant hyphae. In the resistant BM13H roots, the fungus penetrated no further than the endodermis of the main root (the taproot), although it could colonize almost all of the enclosing cortex and epidermal tissues (e.g., Figure 4d). However, in the susceptible YZ roots, fungal hyphae were often detected within the stele, and in the xylem vessels (e.g., Figure 4b). The infection process and single-locus nature of BM13H resistance resembles the single-gene resistance in tobacco identified from *Nicotiana deneyi* Domin [27] called *resistance to BRR 1* (RBRR1), that was transferred to burley tobacco production in the 1950s and has remained a durable source of BRR resistance ever since [28]. Hood and Shew [22] compared BRR infection in a BRR-resistant tobacco cultivar ‘Tennessee 90′ (TN90) containing RBRR1, to a susceptible tobacco cultivar ‘Burley 21 x Kentucky 10′ (B2IxI0). They found that for TN90 spore germination, germ tube growth and penetration of root tissue were similar to B2IxI0 but that hyphal invasion was more rapid and spores formed earlier on B2IxI0 roots, and that invasive hyphae penetrated the stele of B2IxI0 roots but not TN90 roots. Hyphal growth and lesion development were limited in TN90 with sporulation rarely being observed. Due to the limited fungal growth and absence of secondary infections, the root system of TN90 gradually outgrew the effects of the initial inoculation [22]. Our results for BM13H appear to be similar to that observed in tobacco although sporulation may be more common in BM13H than observed in TN90, it appears that BM13H can limit the primary infection and reduce secondary infection such that root growth is not adversely affected, and as older roots appear to be more resistant [3], this allows BM13H to outgrow the disease.

Transcriptome analysis reflected the microscopic observations on the infection process, as BM13H had early gene expression responses at 2 and 3 dpi with infection-specific DEG GO terms being associated with cell wall development, and carbohydrate biosynthesis and metabolic processes, but few DEGs were identified at later time points (Table 2, Figure S2). This lack of DEGs after 5 dpi possibly reflects that BM13H restricts fungal growth such that root growth and development is relatively unaffected at later time points leaving the transcriptomic landscape relatively unchanged. Whereas, the susceptible accession YZ had a very different transcriptional response with a small number of expression changes observed at 2 dpi, but then very large numbers of gene expression changes were observed for evaluations after 5 dpi with YZ infection specific GO terms associated with the regulation of signaling and the immune system, and defense and stress responses (Table 3, Figure S3). The increasing DEG changes probably reflect the increasing level of fungal infection on the roots and cell death occurring in YZ that results in blackened, stunted roots and are similar to the protein expression changes observed during a compatible interaction between *B. basicola* and *G. hirsutum* (Sicot 189) in which 29% and 24% of identified protein expression changes were associated with defense and stress, respectively [29].

Genetic analysis located the RBR01 locus to a 128,488 bp region on chromosome 1, a region annotated for ten genes including five putative resistance-like genes. Of these genes only *Ga01G2665*, *Ga01G2666* and *Ga01G2671* were differentially expressed between infected and uninfected YZ roots at 10 dpi (Figure 7). There were a large number of accession-based gene expression changes observed between uninfected BM13H and YZ roots that may be associated with the resistance phenotype. Three genes in the mapped interval were consistently differentially expressed between uninfected BM13H and YZ roots; *Ga01G2669* and *Ga01G2671*, 3.4- and 2.3-fold higher in BM13H, respectively, and *Ga01G2672* 3-fold higher in YZ (Figure 7). *Ga01G2669* is an uncharacterized protein, whereas *Ga01G2671* and *Ga01G2672* share homology to putative disease resistance proteins. Therefore, resistance may be associated with constitutive expression differences between YZ and BM13H and not infection associated DEGs between the two accessions. As RBR01 resistance is partially dominant the most likely BRR resistance candidate gene based on available information is *Ga01G2671* that is overexpressed in the resistant accession. Although, it must be noted that the genome and transcriptome mapping analyses are dependent on the genome sequence information derived from the *G arboreum* accession SXY1 [16] as de novo genomes assemblies of BM13H and YZ are not available, and it is known in *G. hirsutum* that there can be extensive intraspecific gene order and gene structural variations between cultivars in some regions [30]. In the future, functional confirmation of the candidate genes using a transgenic approach must be performed before the gene/s responsible for resistance can be conclusively determined. However, *G. arboreum* is very difficult to stably transform; thus, this analysis may need to be performed in *G. hirsutum* that is more easily transformed.

Additionally *G. arboreum* is sexually incompatible with *G. hirsutum* due to genome size and structural differences so to transfer this new BRR resistance locus to commercial *G. hirsutum* cultivars will require either a molecular genetics approach via transgenic introgression of the resistance gene or a breeding approach via the formation of a synthetic tetraploid or synthetic hexaploid bridging species that enable sexual crossing to *G. hirsutum* [31,32]. The molecular approaches require knowledge of the causal gene and are potentially quicker, but also assumes that the resistance gene works independently or that the downstream molecular pathways required for the resistance mechanism are also present in *G. hirsutum*; although such disease resistance pathways are likely conserved due to the common origin of the A-genome for this species [33]. The breeding approach has been successful in the introgression of *Rotylenchus reniformis* resistance from the F-genome type *G. longicalyx* to *G. hirsutum* [34]. However, it is a very long process, even with modern high throughput genotyping and genome information, and has proven to be difficult for the introgression of the BRR resistance from *G. herbaceum* ‘A20’ [10]. Nonetheless, the simpler nature of the inheritance of the resistance present in BM13H may make this process easier.

## 4. Materials and Methods

### 4.1. Plant Materials

A population segregating for BRR resistance was created by crossing the highly resistant *G. arboreum* accession BM13H [13], with the susceptible *G. arboreum* accession YZ [35]. An F_2_ population of 263 individuals was developed from a single F_1_ plant. A RIL population was developed from 158 F_2_ lines advanced to the F_6_ generation by single seed descent.

### 4.2. Identification of BRR Isolate, Inoculum Preparation and Inoculation System

The fungal isolate WTB-001 was recovered by Dr S. Allen from a cotton plant displaying severe BRR symptoms in a field at the Australian Cotton Research Institute (Narrabri, NSW, Australia). A single spore culture derived from WTB-001 was isolated and grown on carrot agar [36] for 5–6 weeks at 25 °C on Petri plates (145 × 20 mm). The spores were then washed from the plates with sterile water and mixed with glycerol to a final concentration of 15% and stored at −80 °C. To classify the fungal isolate at the species level, a pure culture of WTB-001 was grown on half-strength potato dextrose (PDA) broth (19.5 g potato dextrose (Oxoid Microbiology Products, Hampshire, UK), distilled water 1 L) incubated at 25 °C for 3 days and DNA was isolated using the technique described by de Beer et al. [37]. The ITS region of the rRNA genes was amplified using the primers ITS1 and ITS4 [14] (Table S1) and compared to the published sequences [1].

For plant disease assays, spores from the single spore culture derived from WTB-001 were washed from the Petri plates with 10 mL of sterile water and blended with a Waring blender (Model HGBTAWTS3, Torrington, CT, USA) on high speed for 30 s. The mixture was calibrated for CFU/mL on half-strength PDA agar (19.5 g potato dextrose (Sigma-Aldrich, St Louis, MI, USA), 14.5 g agar course powder (Ajax FineChem, Wollongong, NSW, Australia), distilled water 1 L) incubated at 25 °C for 3 days. CFU counts represent the number of viable propagules and can be composed of chlamydospores, endoconidia, and mycelia.

BRR disease phenotypes for genetic mapping were assessed based on the following method. Initially, 51 F_1_ plants and the F_2_ population were evaluated to assess the genetics of resistance with more extensive evaluation conducted for the RIL population. Fungal inoculum was added such that there were 250 CFU of spores per gram of soil (60:40 mix of compost and perlite) and made in 20 kg batches mixed in a concrete mixer for 30 s. The inoculated soil was added to polypropylene pots (10 cm diameter, 10 cm length) and 8 seeds per F_6_ line were planted per pot. The lines were grown in a temperature-controlled glasshouse watered daily for 4–5 weeks, with a daytime (16 h) temperature of 21–23 °C and night (8 h) temperature of 18–20 °C. A single assay consisted of at least two replicates of each line (~16 plants per replicate), resistant (BM13H) and susceptible (YZ) parents in both infected and uninfected soil (as a control for soil quality). Pots were randomized with respect to their position the glasshouse for each experiment. After ~25 days the soil was carefully washed from the roots. Disease rating (0–100%) was assessed visually as per Niu et al. [12] based on the percentage of necrotic root tissue. A 50% disease rating denotes that necrosis was observed on half the total root area. Each line was subjected to at least two independent assays and the final disease score for each line was the average of the disease rating across all plants and all assays.

For transcriptome experiments, three 2-week-old plants for each parent that were grown in polypropylene pots (10 cm diameter, 10 cm length) and drenched with 100 mL of solution containing 1 × 10^6^ CFU of fungal inoculum suspension. At designated time points (2, 3, 5, 7 and 10 dpi) the soil was carefully washed from the roots of the plants, and plants were rapidly dissected into roots and hypocotyl tissue. Whole roots tissue was collected and placed in liquid nitrogen, and then stored at −80 °C. Three independent root samples were collected for each time point.

For microscopic examination of infection, seeds were surface sterilized in a solution containing 1% NaOCl and 10% ethanol for 15 min and then rinsed several times in sterile distilled water. Seeds were incubated in Petri dishes (145 × 20 mm) with Murashige and Skoog (MS) medium [38] with 0.8% agar for 2 days at 25 °C. Germinated seedlings free from bacterial and fungal contamination were then removed and root dip-inoculated in either a WTB-001 spore suspension (1 × 10^6^ CFU/mL) or sterile water for 5 min. Excess liquid was removed and the seedlings were transferred to sterile, sealed transparent plastic tissue culture pots (65 mm diameter, 100 mm length) containing semi-solid MS media (0.5% agar). The seedlings were incubated at 25 °C with a 16h photoperiod (30 μmolm^−2^/s^−1^).

At one day intervals after inoculation, 4–8 seedlings were gently removed from the agar and transferred into 70% ethanol or lactic acid before staining and microscopic observation. Root surfaces were examined under a dissecting microscope either fresh or after storage in 70% ethanol. Hand sections from fresh roots, or roots preserved in 70% ethanol, were examined under a compound microscope either unstained or stained with 1% trypan blue.

### 4.3. Plant DNA Sample Preparation and Genotyping

The genomic DNA of the parental *G. arboreum* accessions and their derived F_6_ individuals was extracted from young leaves according to the method described by Ellis et al. [39]. DNA quantity was measured using the NanoDrop ND-1000 spectrophotometer (NanoDrop Technologies, Wilmington, DE, USA) and adjusted to a working concentration of 20 ng/µL. DNA integrity of each sample was checked on a 0.8% agarose gel against Lambda DNA/HindIII size ladder. 

Genotyping of the F_6_ RIL population was performed using multiple methods. KASP markers (Supplementary Table S8) were designed based on SNPs between parental accessions and performed as previously described [39]. SSR and CAPS markers (Supplementary Table S9) were performed as previously described by Lopez-Lavalle et al. [40]. Fluidigm SNPtype™ assays were directed to specific sequences (Table S10) and were performed by Millennium Science Pty Ltd. (Mulgrave, Victoria, Australia) according to the manufacturer’s instructions.

### 4.4. Linkage and QTL Analysis

A linkage map was constructed from 178 polymorphic loci using Joinmap 4.0 software [41]. A LOD score of 3.0 and recombination fraction of 0.4 were used as the threshold criteria for linkage and the Kosambi map function was used to convert recombination frequency to genetic map distance cM [42]. The collinearity of each linkage group was compared to the G. arboreum (SXY1) genome sequence from Du et al. [16] to assist in the assignment of each linkage group. 

QTL analysis was performed using MapQTL5 [17]. Significance thresholds were established by permutation tests (1000 iterations) [43]. The percentage of phenotypic variance explained by the QTL, was calculated at the likelihood peak.

### 4.5. Total RNA Extraction and Transcriptome Sequencing

Total RNA of whole root samples was extracted by Maxwell RSC Plant RNA Kit with the Maxwell RSC Instrument (Promega, Madison, WI, USA). Each sample included three biological replicates. The quality of the RNA samples was checked using the Agilent 2100 Bioanalyzer system (Agilent Technologies, Palo Alto, CA, USA). RNA samples from each time point with an RNA integrity number (RIN) above 7.0 were used for RNA-seq library construction. Libraries were constructed and sequenced by GENEWIZ (Suzhou, China) using the paired-end configuration on an Illumina HiSeq instrument according to the manufacturer’s instructions (Illumina, San Diego, CA, USA). Raw reads were first processed using Trimmomatic v0.39 [44] to remove low-quality sequences and adaptors. The quality of trimmed FASTQ files was evaluated using FastQC v0.11.8. Reads were mapped to the *G. arboreum* reference genome from the cultivar SXY1 [16] using Biokanga version 3.9.8 (https://github.com/csiro-crop-informatics/biokanga, accessed date 17 February 2021) and transcript per million mapped reads (TPM) was calculated for estimating gene expression levels with a custom Python script. HTSeqcount [45] was used to obtain the raw read counts of each gene. The R package DESeq2 was used to identify DEGs [46]. An adjusted *p*-value < 0.05 was used as criterion for the determination of DEGs. The raw RNA-Seq data are available from CSIRO data portal (https://data.csiro.au/dap/landingpage?pid=csiro:47747, accessed date 1 March 2021). Gene ontology (GO) enrichment analysis was performed using agriGO v2.0 based on the default settings [47]. The gene lists for GO analysis were obtained by finding DEGs between all pooled infected BM13H samples versus all YZ infected samples and removing all DEGs between all pooled uninfected BM13H samples versus all YZ uninfected samples. 

## 5. Conclusions

A new resistance locus RBR01 to BRR (*B. rouxiae*) was identified in the *G. arboreum* accession BM13H and mapped to chromosome 1. Analysis of the root infection process revealed that resistance was associated with the slowing down of fungal infection and reduction in sporulation, which enable the infected roots to outgrow the disease. Fine mapping reduced the locus region to 128.5 kbp containing ten annotated genes with the most likely candidate being a putative resistance-like gene *Ga01G2671* that is constitutively more highly expressed in resistant accession BM13H compared to the susceptible accession YZ.

## Figures and Tables

**Table 1:** Number of differentially expressed genes (DEGs) between infected and uninfected root tissue during BRR infection of BM13H and YZ.

	YZ		BM13H	
Days Post Infection	Upregulated	Downregulated	Upregulated	Downregulated
2	3	5	43	10
3	57	15	59	74
5	1606	363	5	3
7	1660	661	0	2
10	3740	2592	2	0

**Table 2:** Gene Ontology (GO) terms enriched in BM13H during infection by *B. rouxiae*.

Description	Ontology	Number of Genes	False Discovery Rate
glucan metabolic process	Biological process	35	1.3 × 10^−5^
cellular glucan metabolic process	Biological process	35	1.3 × 10^−5^
glucan biosynthetic process	Biological process	29	1.6 × 10^−5^
carbohydrate metabolic process	Biological process	84	2.6 × 10^−5^
polysaccharide biosynthetic process	Biological process	34	3.3 × 10^−5^
cellular carbohydrate metabolic process	Biological process	43	3.3 × 10^−5^
cellular polysaccharide metabolic process	Biological process	37	3.3 × 10^−5^
cellular polysaccharide biosynthetic process	Biological process	31	6.4 × 10^−5^
polysaccharide metabolic process	Biological process	41	6.4 × 10^−5^
cellular carbohydrate biosynthetic process	Biological process	32	0.000
starch metabolic process	Biological process	21	0.000
starch biosynthetic process	Biological process	18	0.000
cellular component organization or biogenesis	Biological process	113	0.002
carbohydrate biosynthetic process	Biological process	37	0.003
cell wall organization or biogenesis	Biological process	35	0.004
cellular component organization	Biological process	103	0.005
external encapsulating structure organization	Biological process	26	0.012
single-organism carbohydrate metabolic process	Biological process	53	0.012
maltose metabolic process	Biological process	12	0.012
plant-type cell wall organization or biogenesis	Biological process	22	0.012
cell wall organization	Biological process	25	0.014
plastid organization	Biological process	21	0.019
plant-type cell wall organization	Biological process	17	0.033
extracellular region	Cellular component	39	0.001

**Table 3:** GO terms enriched in YZ during infection by *B. rouxiae.*

Description	Ontology	Number of Genes	False Discovery Rate
defense response	Biological process	68	1.1 × 10^−6^
response to organonitrogen compound	Biological process	26	5.0 × 10^−6^
response to biotic stimulus	Biological process	61	2.7 × 10^−5^
regulation of defense response	Biological process	32	2.7 × 10^−5^
response to chitin	Biological process	23	2.7 × 10^−5^
regulation of response to stress	Biological process	32	2.7 × 10^−5^
response to nitrogen compound	Biological process	34	2.8 × 10^−5^
immune system process	Biological process	44	2.9 × 10^−5^
response to stress	Biological process	138	4.7 × 10^−5^
cell death	Biological process	32	0.000
regulation of innate immune response	Biological process	25	0.000
innate immune response	Biological process	40	0.000
regulation of immune response	Biological process	25	0.000
response to external biotic stimulus	Biological process	54	0.000
regulation of immune system process	Biological process	25	0.000
immune response	Biological process	40	0.000
response to other organism	Biological process	54	0.000
regulation of cell death	Biological process	24	0.000
response to external stimulus	Biological process	70	0.000
regulation of response to stimulus	Biological process	42	0.000
response to stimulus	Biological process	218	0.000
response to oxygen-containing compound	Biological process	78	0.000
response to chemical	Biological process	125	0.000
programmed cell death	Biological process	27	0.001
regulation of programmed cell death	Biological process	22	0.001
negative regulation of cell death	Biological process	15	0.001
negative regulation of programmed cell death	Biological process	15	0.001
host programmed cell death induced by symbiont	Biological process	23	0.002
plant-type hypersensitive response	Biological process	23	0.002
cell communication	Biological process	88	0.002
salicylic acid mediated signaling pathway	Biological process	17	0.002
response to endogenous stimulus	Biological process	63	0.002
cellular response to salicylic acid stimulus	Biological process	17	0.003
response to salicylic acid	Biological process	19	0.003
response to acid chemical	Biological process	48	0.005
cellular response to stress	Biological process	51	0.005
response to fungus	Biological process	24	0.005
regulation of plant-type hypersensitive response	Biological process	18	0.006
regulation of cellular response to stress	Biological process	18	0.006
response to organic substance	Biological process	82	0.007
cellular response to acid chemical	Biological process	26	0.008
respiratory burst involved in defense response	Biological process	10	0.009
endoplasmic reticulum unfolded protein response	Biological process	12	0.009
cellular response to unfolded protein	Biological process	12	0.009
cellular response to topologically incorrect protein	Biological process	12	0.009
response to unfolded protein	Biological process	12	0.009
multi-organism process	Biological process	61	0.009
signal transduction	Biological process	75	0.010
respiratory burst	Biological process	10	0.010
protein targeting to membrane	Biological process	18	0.010
single organism signaling	Biological process	75	0.015
signaling	Biological process	75	0.016
response to organic cyclic compound	Biological process	27	0.025
protein localization to membrane	Biological process	18	0.026
establishment of protein localization to membrane	Biological process	18	0.026
cellular response to stimulus	Biological process	98	0.036
defense response to other organism	Biological process	33	0.046
phosphotransferase activity, alcohol group as acceptor	Molecular function	93	0.001
kinase activity	Molecular function	103	0.003
protein kinase activity	Molecular function	81	0.003
protein serine/threonine kinase activity	Molecular function	60	0.017
catalytic activity	Molecular function	471	0.017

## Data Availability

The raw paired-end Illumina RNA sequencing reads generated in the current study are available from the CSIRO data portal https://data.csiro.au/dap/landingpage?pid=csiro:47747 accessed date 1 March 2021.

## Supplementary Materials

Supplementary Materials can be found at https://www.mdpi.com/1422-0067/22/5/2642/s1.
