# Supplementary material for: Characterization and Genetic Mapping of Black Root Rot Resistance in Gossypium arboreum L
Source: Int J Mol Sci. 2021 Mar 5;22(5):2642. doi: 10.3390/ijms22052642 (PMC7961528; doi:10.3390/ijms22052642)
Supplement: Supplementary file 1 [file ijms-22-02642-s001.zip › Supp Figures.pptx]

## Slide 1
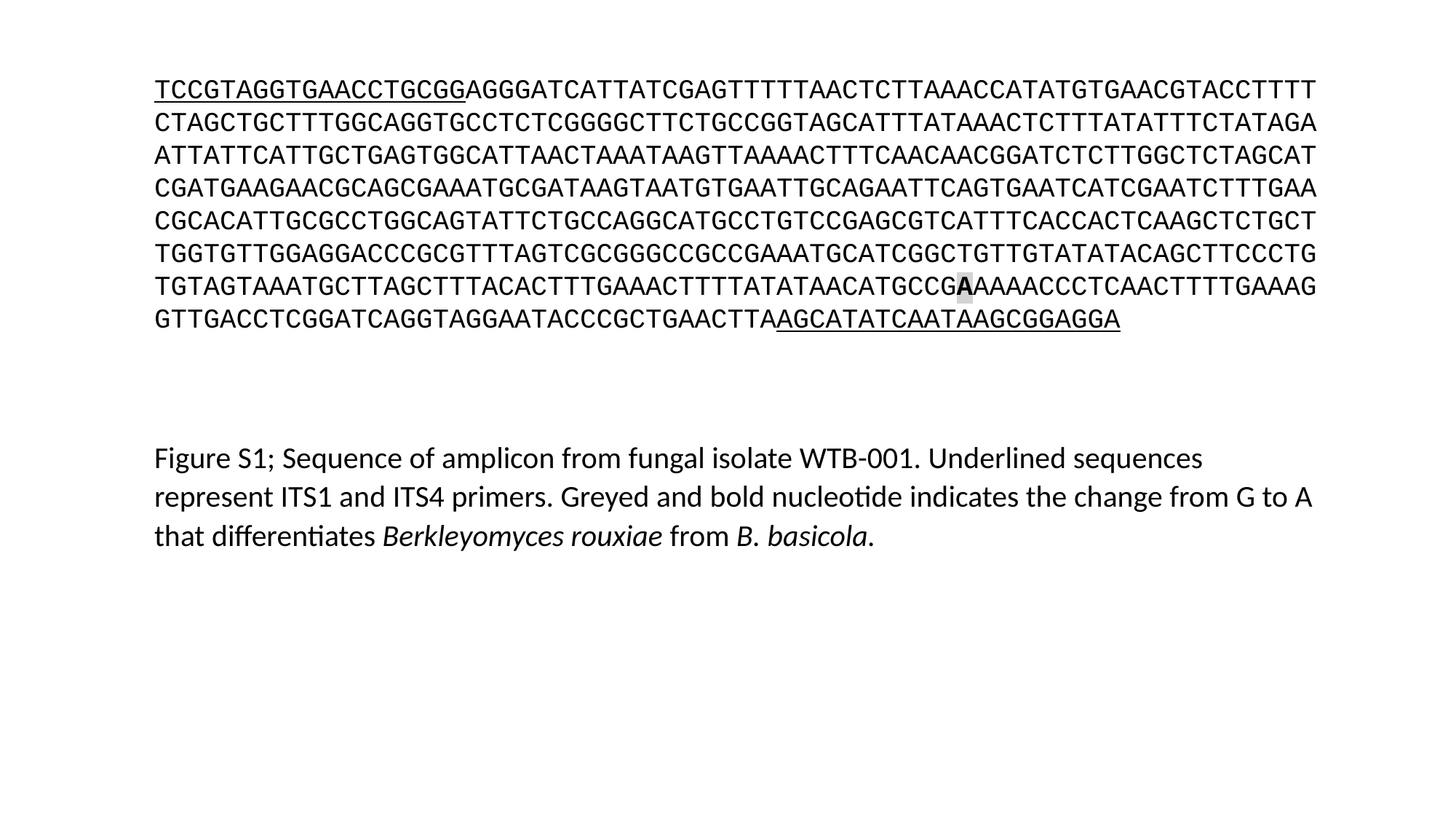

TCCGTAGGTGAACCTGCGGAGGGATCATTATCGAGTTTTTAACTCTTAAACCATATGTGAACGTACCTTTTCTAGCTGCTTTGGCAGGTGCCTCTCGGGGCTTCTGCCGGTAGCATTTATAAACTCTTTATATTTCTATAGAATTATTCATTGCTGAGTGGCATTAACTAAATAAGTTAAAACTTTCAACAACGGATCTCTTGGCTCTAGCATCGATGAAGAACGCAGCGAAATGCGATAAGTAATGTGAATTGCAGAATTCAGTGAATCATCGAATCTTTGAACGCACATTGCGCCTGGCAGTATTCTGCCAGGCATGCCTGTCCGAGCGTCATTTCACCACTCAAGCTCTGCTTGGTGTTGGAGGACCCGCGTTTAGTCGCGGGCCGCCGAAATGCATCGGCTGTTGTATATACAGCTTCCCTGTGTAGTAAATGCTTAGCTTTACACTTTGAAACTTTTATATAACATGCCGAAAAACCCTCAACTTTTGAAAGGTTGACCTCGGATCAGGTAGGAATACCCGCTGAACTTAAGCATATCAATAAGCGGAGGA
Figure S1; Sequence of amplicon from fungal isolate WTB-001. Underlined sequences represent ITS1 and ITS4 primers. Greyed and bold nucleotide indicates the change from G to A that differentiates Berkleyomyces rouxiae from B. basicola.

## Slide 2
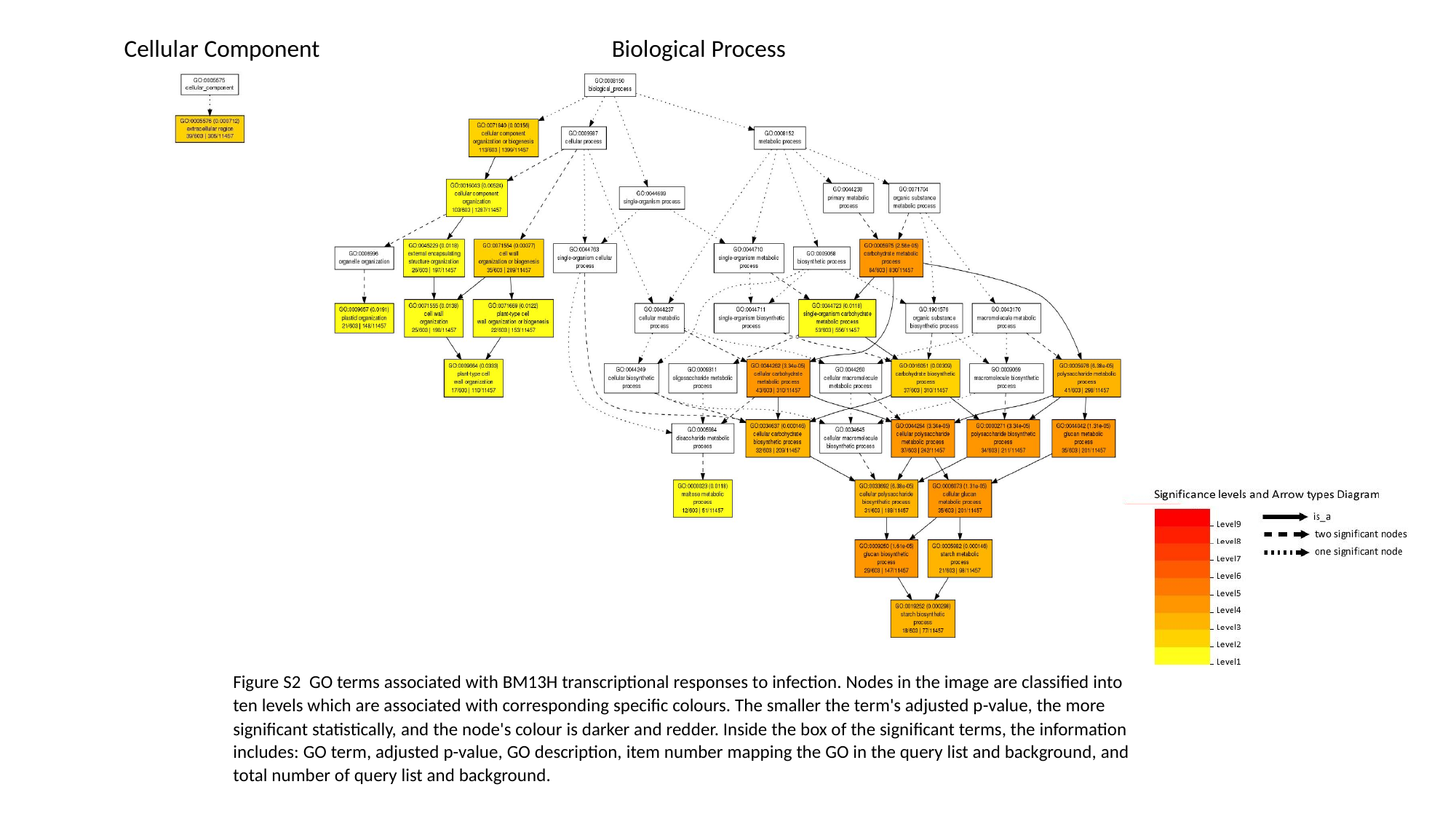

Cellular Component
Biological Process
Figure S2 GO terms associated with BM13H transcriptional responses to infection. Nodes in the image are classified into ten levels which are associated with corresponding specific colours. The smaller the term's adjusted p-value, the more significant statistically, and the node's colour is darker and redder. Inside the box of the significant terms, the information includes: GO term, adjusted p-value, GO description, item number mapping the GO in the query list and background, and total number of query list and background.

## Slide 3
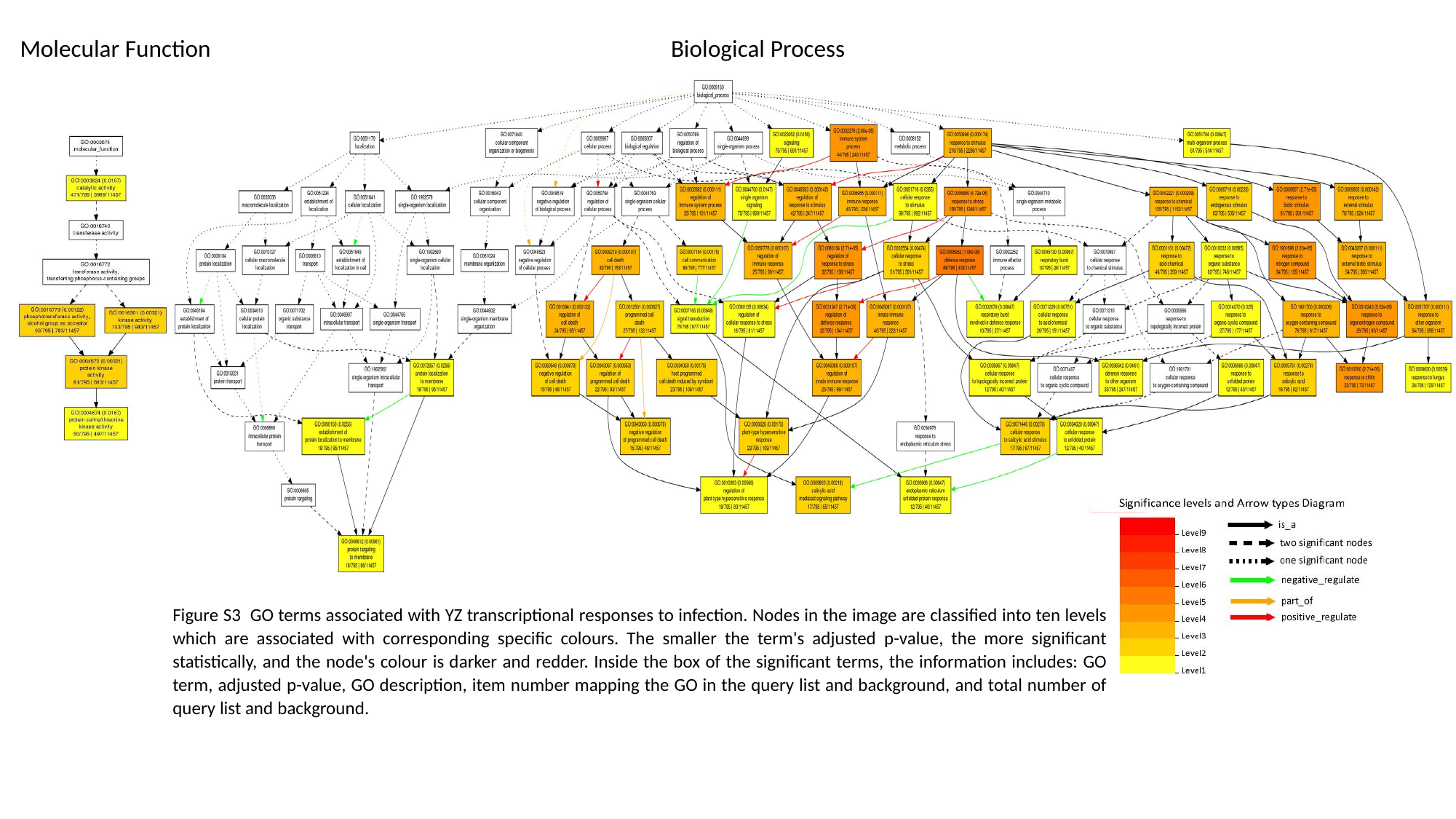

Molecular Function
Biological Process
Figure S3 GO terms associated with YZ transcriptional responses to infection. Nodes in the image are classified into ten levels which are associated with corresponding specific colours. The smaller the term's adjusted p-value, the more significant statistically, and the node's colour is darker and redder. Inside the box of the significant terms, the information includes: GO term, adjusted p-value, GO description, item number mapping the GO in the query list and background, and total number of query list and background.

## Slide 4
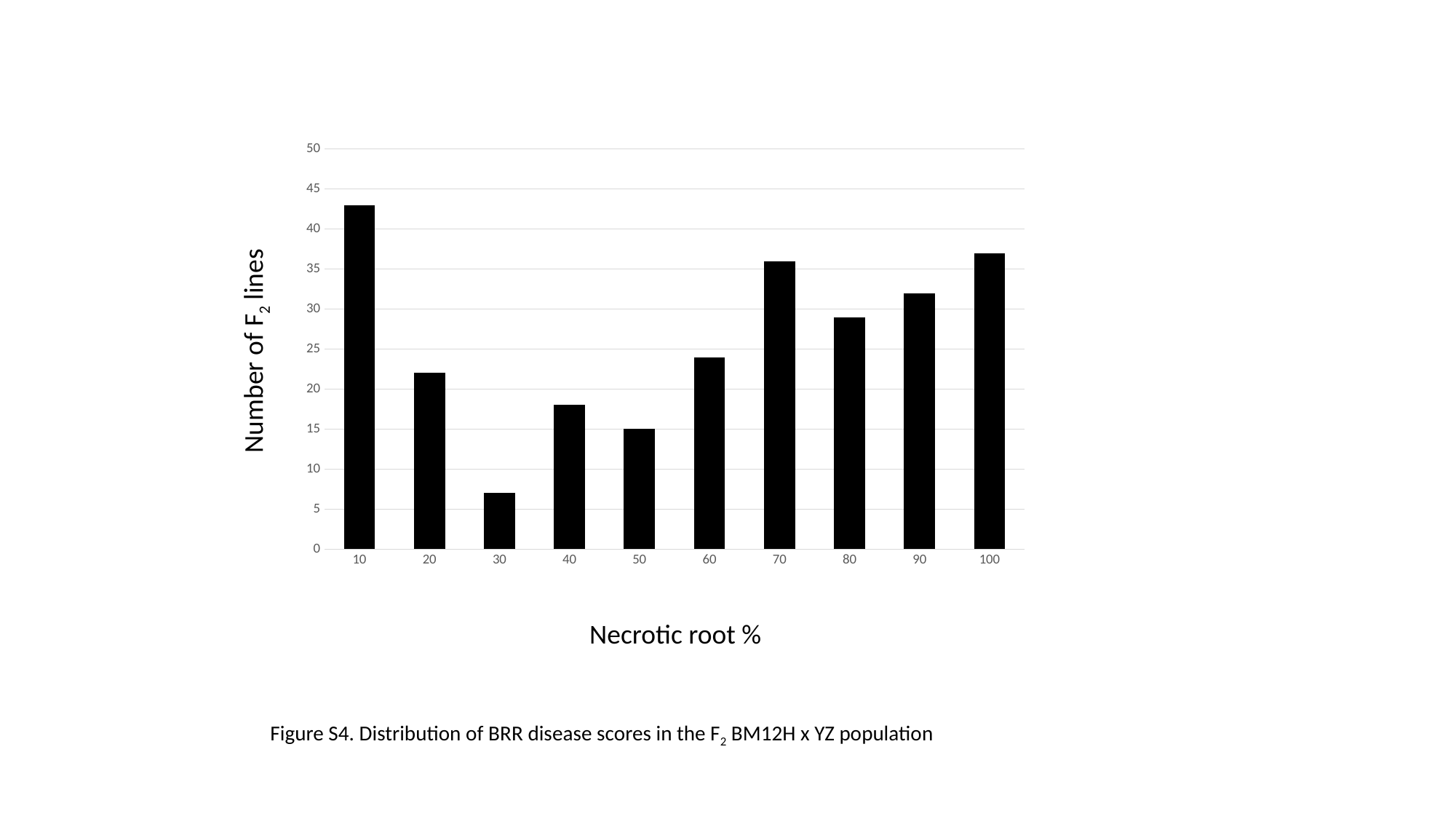

### Chart
| Category | |
|---|---|
| 10 | 43.0 |
| 20 | 22.0 |
| 30 | 7.0 |
| 40 | 18.0 |
| 50 | 15.0 |
| 60 | 24.0 |
| 70 | 36.0 |
| 80 | 29.0 |
| 90 | 32.0 |
| 100 | 37.0 |Number of F2 lines
Necrotic root %
Figure S4. Distribution of BRR disease scores in the F2 BM12H x YZ population

## Slide 5
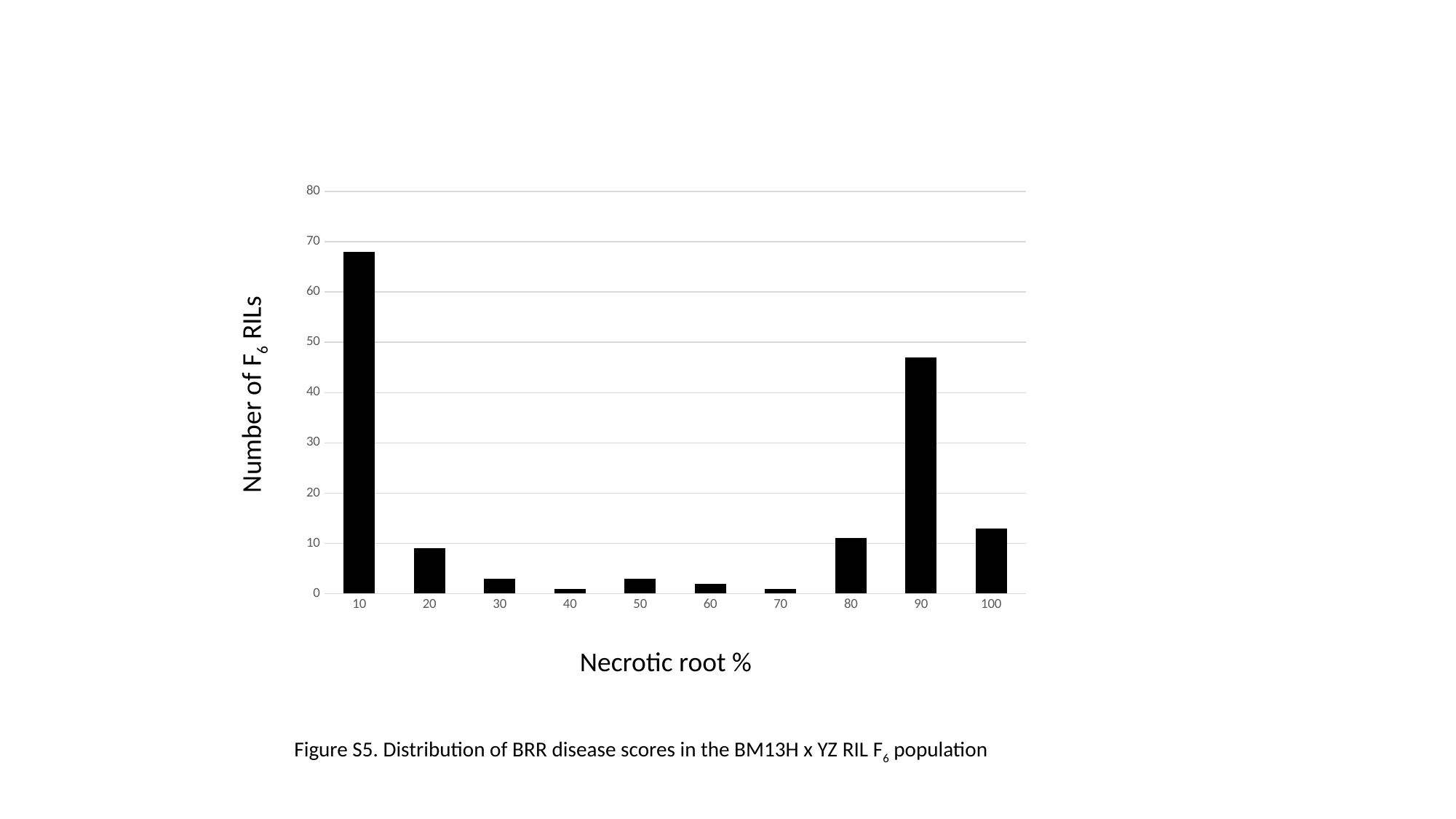

### Chart
| Category | |
|---|---|
| 10 | 68.0 |
| 20 | 9.0 |
| 30 | 3.0 |
| 40 | 1.0 |
| 50 | 3.0 |
| 60 | 2.0 |
| 70 | 1.0 |
| 80 | 11.0 |
| 90 | 47.0 |
| 100 | 13.0 |Number of F6 RILs
Necrotic root %
Figure S5. Distribution of BRR disease scores in the BM13H x YZ RIL F6 population

## Slide 6
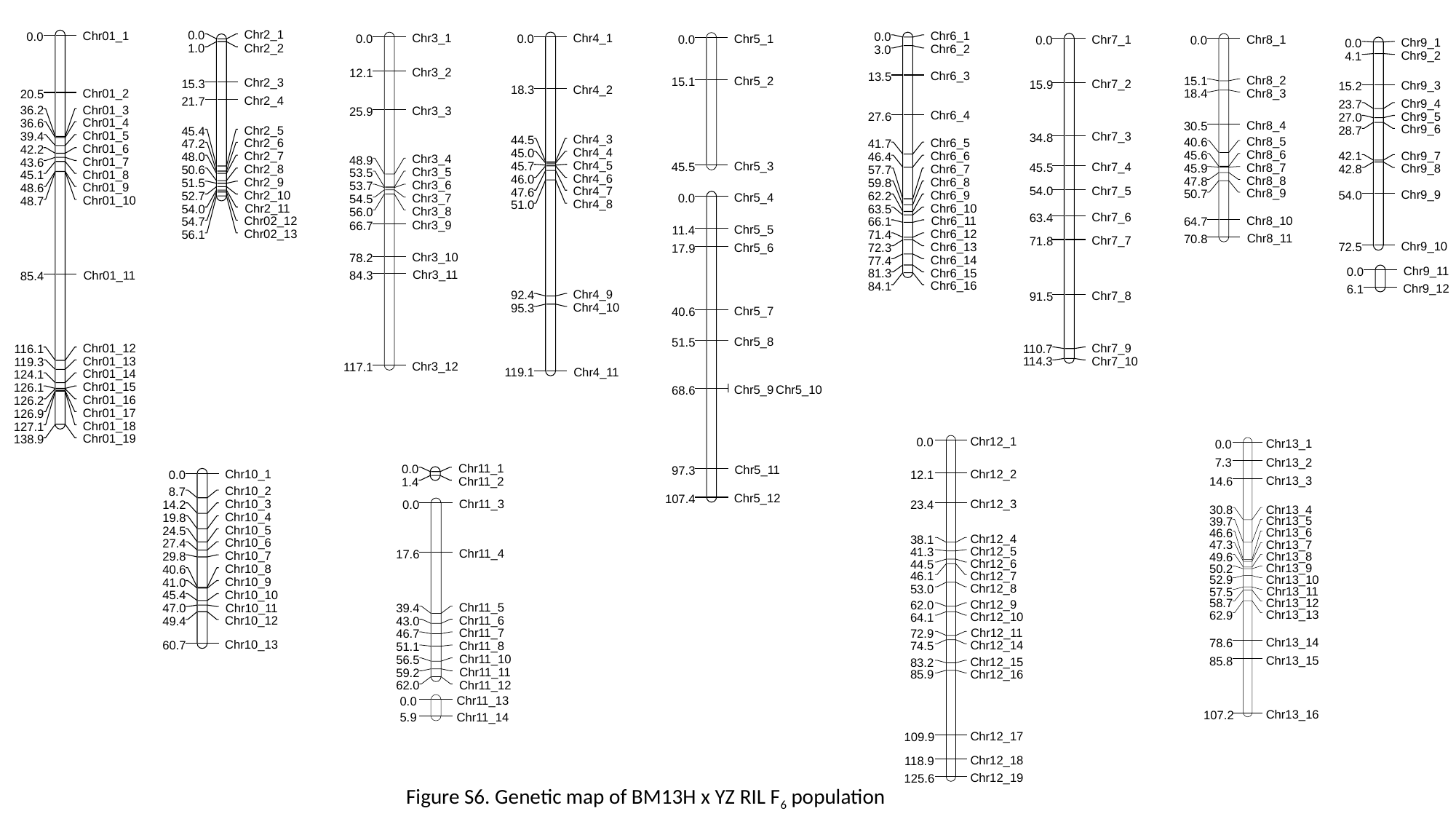

Chr2_1
0.0
Chr2_2
1.0
Chr2_3
15.3
Chr2_4
21.7
Chr2_5
45.4
Chr2_6
47.2
Chr2_7
48.0
Chr2_8
50.6
Chr2_9
51.5
Chr2_10
52.7
Chr2_11
54.0
Chr02_12
54.7
Chr02_13
56.1
Chr6_1
0.0
Chr6_2
3.0
Chr6_3
13.5
Chr6_4
27.6
Chr6_5
41.7
Chr6_6
46.4
Chr6_7
57.7
Chr6_8
59.8
Chr6_9
62.2
Chr6_10
63.5
Chr6_11
66.1
Chr6_12
71.4
Chr6_13
72.3
Chr6_14
77.4
Chr6_15
81.3
Chr6_16
84.1
Chr01_1
0.0
Chr01_2
20.5
Chr01_3
36.2
Chr01_4
36.6
Chr01_5
39.4
Chr01_6
42.2
Chr01_7
43.6
Chr01_8
45.1
Chr01_9
48.6
Chr01_10
48.7
Chr01_11
85.4
Chr01_12
116.1
Chr01_13
119.3
Chr01_14
124.1
Chr01_15
126.1
Chr01_16
126.2
Chr01_17
126.9
Chr01_18
127.1
Chr01_19
138.9
Chr4_1
0.0
Chr4_2
18.3
Chr4_3
44.5
Chr4_4
45.0
Chr4_5
45.7
Chr4_6
46.0
Chr4_7
47.6
Chr4_8
51.0
Chr4_9
92.4
Chr4_10
95.3
Chr4_11
119.1
Chr3_1
0.0
Chr3_2
12.1
Chr3_3
25.9
Chr3_4
48.9
Chr3_5
53.5
Chr3_6
53.7
Chr3_7
54.5
Chr3_8
56.0
Chr3_9
66.7
Chr3_10
78.2
Chr3_11
84.3
Chr3_12
117.1
Chr5_1
0.0
Chr5_2
15.1
Chr5_3
45.5
Chr5_4
0.0
Chr5_5
11.4
Chr5_6
17.9
Chr5_7
40.6
Chr5_8
51.5
Chr5_9
Chr5_10
68.6
Chr5_11
97.3
Chr5_12
107.4
Chr7_1
0.0
Chr7_2
15.9
Chr7_3
34.8
Chr7_4
45.5
Chr7_5
54.0
Chr7_6
63.4
Chr7_7
71.8
Chr7_8
91.5
Chr7_9
110.7
Chr7_10
114.3
Chr8_1
0.0
Chr8_2
15.1
Chr8_3
18.4
Chr8_4
30.5
Chr8_5
40.6
Chr8_6
45.6
Chr8_7
45.9
Chr8_8
47.8
Chr8_9
50.7
Chr8_10
64.7
Chr8_11
70.8
Chr9_1
0.0
Chr9_2
4.1
Chr9_3
15.2
Chr9_4
23.7
Chr9_5
27.0
Chr9_6
28.7
Chr9_7
42.1
Chr9_8
42.8
Chr9_9
54.0
Chr9_10
72.5
Chr9_11
0.0
Chr9_12
6.1
Chr12_1
0.0
Chr12_2
12.1
Chr12_3
23.4
Chr12_4
38.1
Chr12_5
41.3
Chr12_6
44.5
Chr12_7
46.1
Chr12_8
53.0
Chr12_9
62.0
Chr12_10
64.1
Chr12_11
72.9
Chr12_14
74.5
Chr12_15
83.2
Chr12_16
85.9
Chr12_17
109.9
Chr12_18
118.9
Chr12_19
125.6
Chr13_1
0.0
Chr13_2
7.3
Chr13_3
14.6
Chr13_4
30.8
Chr13_5
39.7
Chr13_6
46.6
Chr13_7
47.3
Chr13_8
49.6
Chr13_9
50.2
Chr13_10
52.9
Chr13_11
57.5
Chr13_12
58.7
Chr13_13
62.9
Chr13_14
78.6
Chr13_15
85.8
Chr13_16
107.2
Chr11_1
0.0
Chr11_2
1.4
Chr11_3
0.0
Chr11_4
17.6
Chr11_5
39.4
Chr11_6
43.0
Chr11_7
46.7
Chr11_8
51.1
Chr11_10
56.5
Chr11_11
59.2
Chr11_12
62.0
Chr11_13
0.0
Chr11_14
5.9
Chr10_1
0.0
Chr10_2
8.7
Chr10_3
14.2
Chr10_4
19.8
Chr10_5
24.5
Chr10_6
27.4
Chr10_7
29.8
Chr10_8
40.6
Chr10_9
41.0
Chr10_10
45.4
Chr10_11
47.0
Chr10_12
49.4
Chr10_13
60.7
Figure S6. Genetic map of BM13H x YZ RIL F6 population
